# Gallbladder agenesis a rare and underdiagnosed congenital anomaly: a case report and literature review

**DOI:** 10.1093/jscr/rjac505

**Published:** 2022-11-10

**Authors:** Rodrigo Piltcher-da-Silva, Vivian Laís Sasaki, Dóroty Eva Garcia Felisberto, Beatriz Carolina Schuta Bodanese, Mariana Piltcher-Recuero, Bianca Vitória Schuta Bodanese, Luiz Francisco Cravo Bettini, Yan Sacha Hass Aguilera, Marco Raeder da Costa, Júlio Cezar Uili Coelho

**Affiliations:** General and Digestive Surgery Service, Hospital Nossa Senhora das Graças, Curitiba, Brazil; General and Digestive Surgery Service, Hospital Nossa Senhora das Graças, Curitiba, Brazil; General and Digestive Surgery Service, Hospital Nossa Senhora das Graças, Curitiba, Brazil; General and Digestive Surgery Service, Hospital Nossa Senhora das Graças, Curitiba, Brazil; General and Digestive Surgery Service, Hospital Nossa Senhora das Graças, Curitiba, Brazil; General and Digestive Surgery Service, Hospital Nossa Senhora das Graças, Curitiba, Brazil; General and Digestive Surgery Service, Hospital Nossa Senhora das Graças, Curitiba, Brazil; General and Digestive Surgery Service, Hospital Nossa Senhora das Graças, Curitiba, Brazil; General and Digestive Surgery Service, Hospital Nossa Senhora das Graças, Curitiba, Brazil; General and Digestive Surgery Service, Hospital Nossa Senhora das Graças, Curitiba, Brazil

## Abstract

Gallbladder agenesis (GA) is a rare congenital anomaly with conflicting epidemiology described in the literature. When present, it is misinterpreted as cholelitiasis, a highly prevalent condition. Nevertheless, surgeons and radiologists must be aware of it since it can lead to unnecessary invasive procedures. Diagnosis of GA is challenging due to the anatomical structures that sometimes resemble a shrunken gallbladder. We report the case of a 55-year-old man with preoperative diagnosis of cholelitiasis and further intraoperative find of GA. Since cholecystectomy is one of the most common surgeries worldwide, it demonstrates how relevant this case is to emphasize the need to recognize this diagnosis and be aware of its management to avoid unnecessary surgery.

## INTRODUCTION

The prevalence of cholelithiasis is 10% in most western countries. and its treatment is cholecystectomy, which is the most common abdominal operation worldwide [1]. Gallbladder agenesis (GA) is a rare and underdiagnosed congenital abnormality with prevalence of ~0.04–0.1% [2–6]. It affects females more than males in a proportion of 3:1 [1, 2].

Half of the patients diagnosed with GA refer upper abdominal symptoms, such as biliary colic, nausea, vomiting and dyspepsia. Abdominal ultrasonography (AUS) has a sensitivity of 97% for the diagnosis cholelithiasis. However, its sensitivity decreases to 61% in cases of GA [6]. The duodenum or other intestinal segment usually occupies the gallbladder site in patients with GA, which may give the appearance of gallstone in patients with GA. In this situation, the ultrasound (US) operator expectation of the gallbladder presence in a patient who did not underwent cholecystectomy may result in an erroneous report of cholelithiasis, cholecystitis or even shrunken gallbladder [6]. Thus, the correct diagnosis of GA is commonly done during an unnecessary operation [7].

We present a case of GA in a 55-year-old patient with a history of no previous abdominal operation and an 8-month complaint of abdominal pain, nausea and weight loss. Although the preoperative US diagnosis was chronic scleroatrophic calculous cholecystitis, the gallbladder was absent at laparoscopic exploration of the abdomen.

## PRESENTATION OF CASE

A 55-year-old man presented with an 8-month history of upper abdominal pain associated with nausea, diarrhea and weight loss of 6 lb. At evaluation, he was stable and had pain at abdominal palpation. He had a past medical history of well-controlled hypertension with losartan and no intra-abdominal operation. There was no relevant family history.

**Figure 1:**
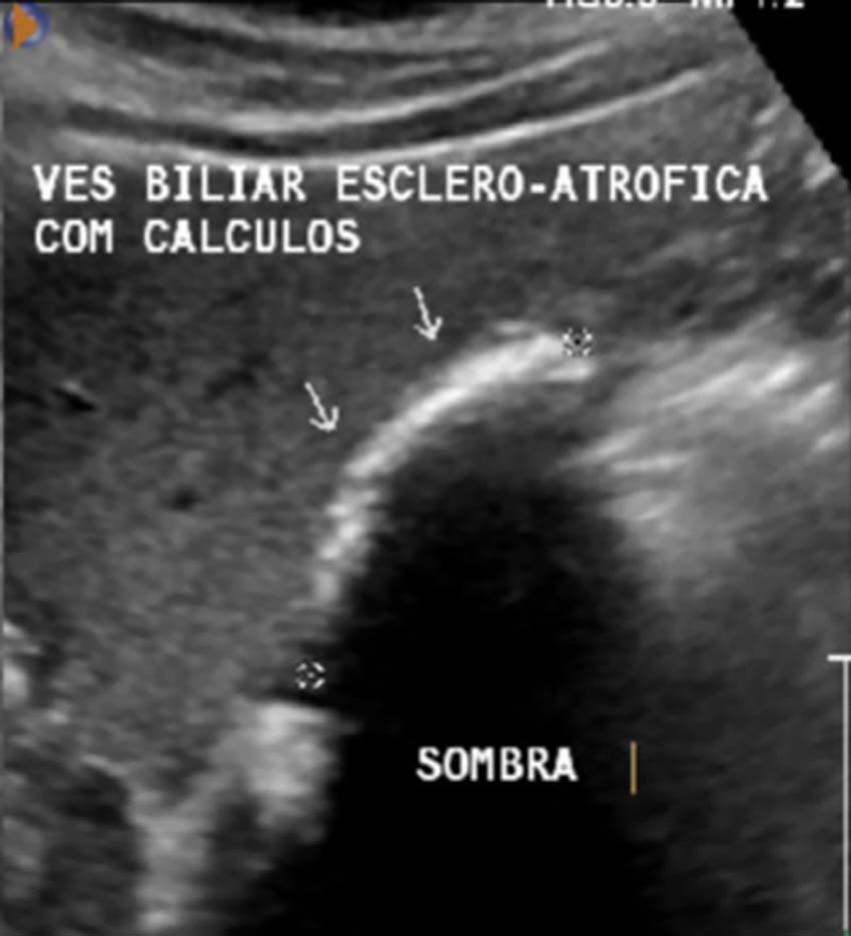
AUS showing a contracted and shrunken gallbladder with gallstones (arrows).

Laboratory tests were uneventful. A US showed a gallbladder with 38 mm in length, scleroatrophic appearance and full of gallstones (Fig. 1). Upper digestive endoscopy showed moderate erosive pangastritis.

**Figure 2:**
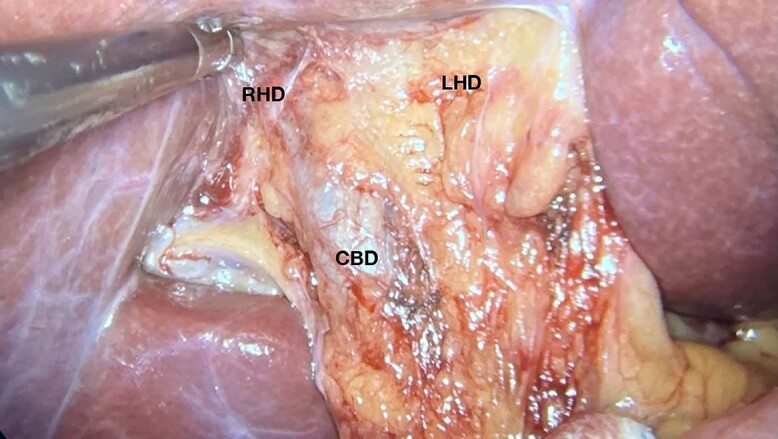
Image showing the gallbladder anatomical site and hepatic hilum without normal nor shrunken gallbladder; CBD, common bile duct; RHD, right hepatic duct; LHD, left hepatic duct.

**Figure 3:**
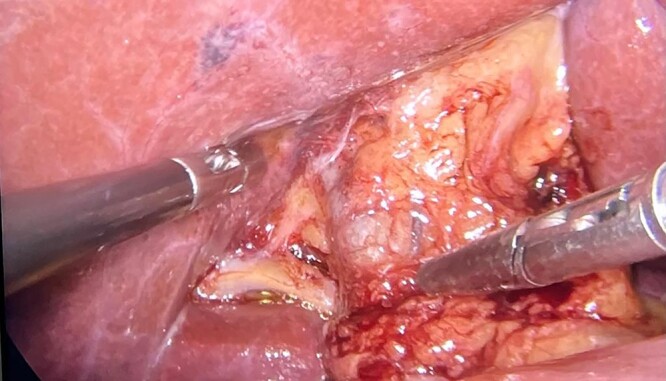
Image showing the hepatic hilum during surgery.

The patient underwent videolaparoscopy in order to perform cholecystectomy, however, no gallbladder was identified in its anatomical site. The common bile duct and hepatic artery were easily dissected and neither the cystic duct nor the cystic artery were identified (Figs 2 and 3). The patient was discharged on the same day of surgery.

**Figure 4:**
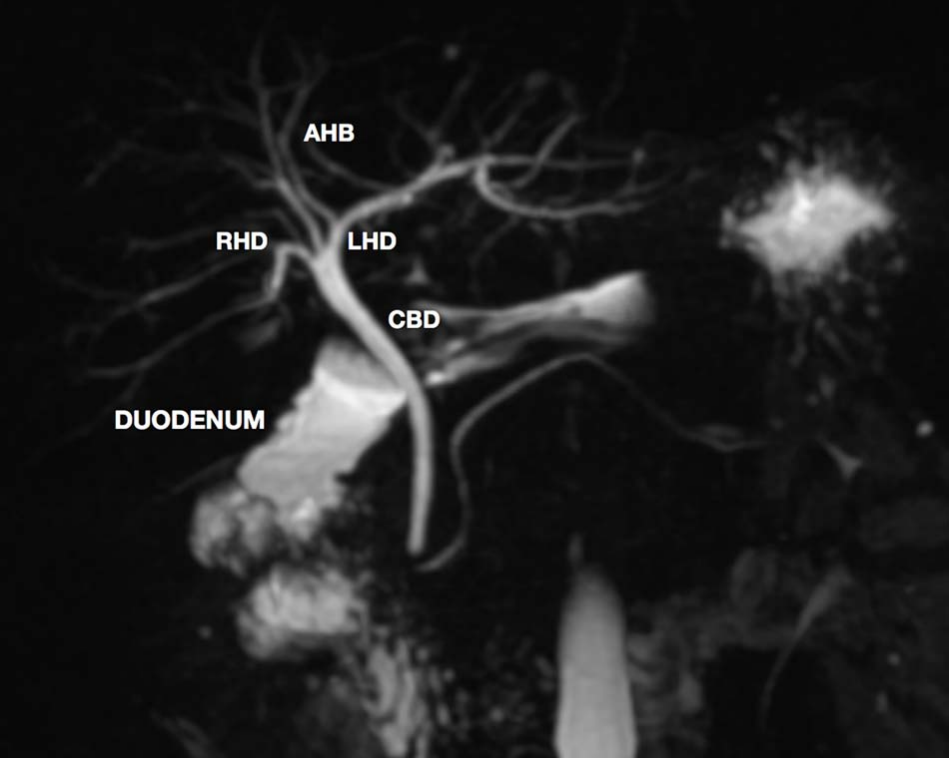
MRCP showing biliary tract with GA and no other biliary anomaly; duodenum and the main pancreatic duct are also with visibility; AHB, anterior hepatic branch.

**Figure 5:**
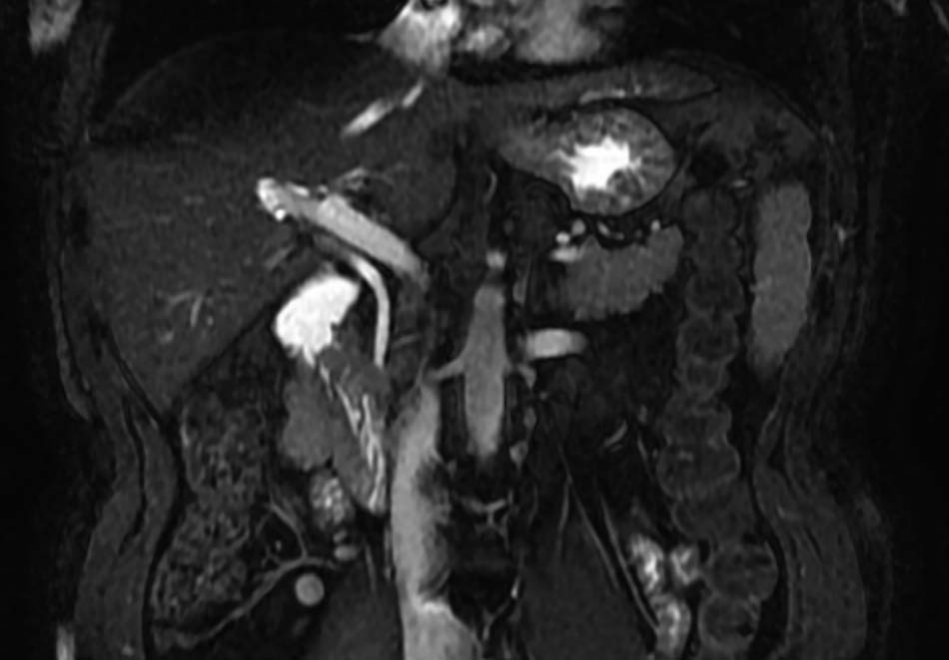
MRCP coronal image showing the common bile duct, portal vein, duodenum, liver and other close structures; there is no sign of gallbladder.

**Figure 6:**
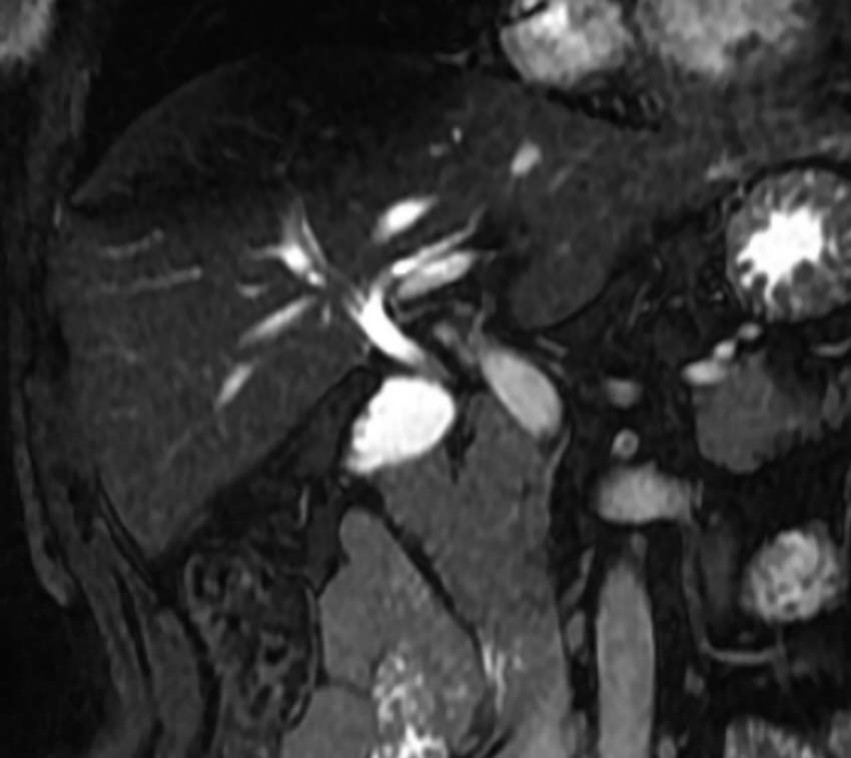
MRCP coronal image showing the intrahepatic biliary tract, with good vision of common bile duct, right and left hepatic ducts and anterior branch duct.

A magnetic resonance cholangiopancreatography (MRCP) confirmed the absence of gallbladder and of the cystic duct and artery (Figs 4–6). No other abdominal anomaly was identified.

## DISCUSSION

Since the first case described by Lemery in 1701 [2, 5], only a few cases of GA have been reported in the literature, mainly in autopsy studies. The gallbladder derives from the hepatic diverticulum in the fourth week of embryonic development, which represents the primordium of the liver. The cystic duct and gallbladder will develop a lumen in the vacuolation process during the seventh week [4].

The pathogenesis of this anomaly is unclear and there are two theories [6]. The first suggests a failure of development of the hepatic diverticulum bud of the foregut, resulting in the malformation [2]. The second theory proposes that an inaccuracy in the process of canalization of the cystic duct and gallbladder may be the cause [2].

GA can be associated with other congenital abnormalities, such as ventricular septal defect, imperforate anus, pancreas divisum, right hepatic lobe hypoplasia and renal agenesis. In 9% of the cases, anomalies of the biliary tract, like atresia of the bile ducts and choledochal cyst, can also be found [8, 9].

It is such a diagnostic challenge because of its poor symptomatology, with 35% of diagnosis being made intraoperative or at autopsy. In the case presented, the patient had symptoms of biliary colic, which represent 50% of the manifestations of GA in symptomatic patients [4–6]. Nausea and vomiting, dyspepsia and jaundice can also occur [6]. These symptoms can derive from biliary stasis and dyskinesia plus elevating the basal pressure of the Oddi sphincter, causing biliary colic [6, 9, 10]. About 35% of patients are asymptomatic and 15% have other fetal anomalies [4, 5].

US imaging is highly sensitive (95%) for cholelitiasis, and it is cost-effective and non-invasive. However, it is operator-dependent and the sensitivity decreases for GA due to the findings mimicking other diseases of the gallbladder [4, 6]. The subhepatic peritoneal folds at what would be the gallbladder bed in the liver can be misinterpreted as a contracted and shrunken gallbladder [4, 5], while the gas-filled duodenum with reflective food content can mimic a stone-filled GB [4, 8]. Thus, due to US image findings and occurrence of abdominal symptoms, surgical treatment is indicated [2].

MRCP is the best exam for the diagnosis of GA since it is non-invasive and allows detailed evaluation of the biliary tract [2]. If the diagnosis of GA is made intraoperative, MRCP is still mandatory to rule out ectopic gallbladder by visualizing the intrahepatic, retroduodenal, retropancreatic and retroperitoneal sites [4]. The risk of inadvertent biliary injury versus a post-operative MRCP does not justify a full surgical exploration, as was advocated in the past [4, 6]. Another option is intraoperative cholangiography to better identify the biliary anatomy [7, 9].

Our case is an example of a typical preoperative misdiagnosis. In order to avoid unnecessary operation, some authors suggest that MRCP may be performed in patients in which the gallbladder is not identified or is scleroatrophic at the AUS. [4]. However, this recommendation is very difficult to be implemented, mainly in developing countries like Brazil, due the high prevalence of cholelithiasis and scarce occurrence of GA.

## CONCLUSION

Since cholelitiasis is a high prevalent condition, GA may be an intraoperative finding in the surgeon’s routine. It is important to consider the diagnosis of GA in patients with no previous abdominal operation in which the gallbladder was not identified. Extensive surgical dissection of the liver hilum should be avoided. MRCP should be performed to exclude ectopic gallbladder.

## ETHICS APPROVAL

This study complies with institutional/national ethical standards. There is no need for evaluation by National Research Ethics Commission to case report.

## CONFLICT OF INTEREST STATEMENT

The authors declare that they have no conflict of interest and that the ethical principles were followed.

## FUNDING

This study did not receive any specific grant from funding agencies in the public, commercial or non-profit sectors.
